# Neural and psychosocial contributions to sex differences in knee osteoarthritic pain

**DOI:** 10.1186/2042-6410-3-26

**Published:** 2012-12-17

**Authors:** Kathleen A Sluka, Karen J Berkley, Mary I O’Connor, Daniel P Nicolella, Roger M Enoka, Barbara D Boyan, David A Hart, Eileen Resnick, C Kent Kwoh, Laura L Tosi, Richard D Coutts, Wendy M Kohrt

**Affiliations:** 1 Isis Research Network on Musculoskeletal Health, Iowa City, USA; 2 Physical Therapy and Rehabilitation Science, 1–252 Medical Education Building, University of Iowa, 52242-1190, Iowa City, IA, USA

**Keywords:** Osteoarthritis, Pain, Sex differences, Gender, Nociceptor, Central sensitization, Psychosocial, Catastrophizing

## Abstract

People with osteoarthritis (OA) can have significant pain that interferes with function and quality of life. Women with knee OA have greater pain and greater reductions in function and quality of life than men. In many cases, OA pain is directly related to sensitization and activation of nociceptors in the injured joint and correlates with the degree of joint effusion and synovial thickening. In some patients, however, the pain does not match the degree of injury and continues after removal of the nociceptors with a total joint replacement. Growth of new nociceptors, activation of nociceptors in the subchondral bone exposed after cartilage degradation, and nociceptors innervating synovium sensitized by inflammatory mediators could all augment the peripheral input to the central nervous system and result in pain. Enhanced central excitability and reduced central inhibition could lead to prolonged and enhanced pain that does not directly match the degree of injury. Psychosocial variables can influence pain and contribute to pain variability. This review explores the neural and psychosocial factors that contribute to knee OA pain with an emphasis on differences between the sexes and gaps in knowledge.

## Review

### Introduction

The International Association for the Study of Pain (IASP) defines pain as an unpleasant sensory and emotional experience associated with actual or potential tissue damage, or described in terms of such damage. Pain, therefore, is a subjective experience that not only involves the sensation itself, but also includes an unpleasantness dimension to the experience (see Table 1 for pain terminology). The pain associated with osteoarthritis (OA) can limit function and reduce quality of life. Although OA pain may appear to reflect activation of nociceptors in the damaged joint in some cases, what determines the actual perception of pain at any time is how the central nervous system (CNS) uses and modifies information derived not only from nociceptors but also from other sources. (The IASP defines a nociceptor as “A high-threshold sensory receptor of the peripheral somatosensory nervous system that is capable of transducing and encoding noxious stimuli.”). The mechanisms underlying these differences in pain between women and men with knee OA are unknown. Improving our understanding of the mechanisms underlying sex differences in the perception of pain in OA will likely lead to more effective, and possibly sex-specific, treatment strategies.

**Table 1:** **Pain terminology as defined by the International Association for the Study of Pain (**http://www.iasp-pain.org**)**

**Terminology**	**Definition**	**Comments**
*Nociceptor*	A sensory receptor that is capable of transducing and encoding a noxious stimulus	Nociceptors are located in a variety of tissue types including most structures of the articular joint
*Nociceptive neuron*	A central or peripheral neuron that is capable of encoding noxious stimulation	
*Sensitization*	Increased responsiveness of neurons to normal input or activation of a response by inputs that are normally subthreshold	Can occur in the periphery (nociceptors) or in the CNS pathway
*Primary hyperalgesia*	Increased response to a stimulus that is normally painful	Occurs at the site of insult; results from peripheral nociceptor sensitization
*Secondary hyperalgesia*	Increased response to a stimulus that is normally painful	Occurs outside the site of insult in tissue that is not injured; results from central neuron sensitization
*Allodynia*	Pain due to a stimulus that does not normally evoke pain	Results from central neuron sensitization
*Temporal Summation*	Increased pain to application of the same stimulus repetitively over a brief period of time	Enhanced in patients with osteoarthritis; surrogate measure of central neuron excitability
*Conditioned pain modulation*	Application of a noxious stimulus distant to the test area (such as leg) produces analgesia (such as arm)	Decreased in patients with osteoarthritis; surrogate measure of central inhibition; also referred to as diffuse noxious inhibitory control (DNIC)
*Pain catastrophizing*	Tendency to magnify the consequences of the pain and to feel helpless in managing the pain	Higher levels associated with higher OA pain, and worse outcomes (disability, pain)
*Self-efficacy*		Lower levels are associated with worse outcomes (function, pain)
*Fear of pain/movement*	Excessive expectation that physical activity will worsen pain and function	Higher levels are associated with worse outcomes (function, disability, pain)

Significant clinical differences in pain exist between men and women with knee osteoarthritis. Women more commonly have OA of the knee and lose articular cartilage from the proximal tibia at four times the annual rate of men and from the patella at three times a greater rate [1]. In patients with knee OA who undergo total knee arthroplasty, women have lower functional scores and report greater pain than men both before and after total knee arthroplasty [1]. In a large study of 5290 patients two years after surgery, 36% more women than men had moderate to severe pain at 2 years [2]. Even with adjustment for age and preoperative pain level, women were more likely to have moderate-to-severe pain at 2 years after arthroplasty.

**Figure 1:**
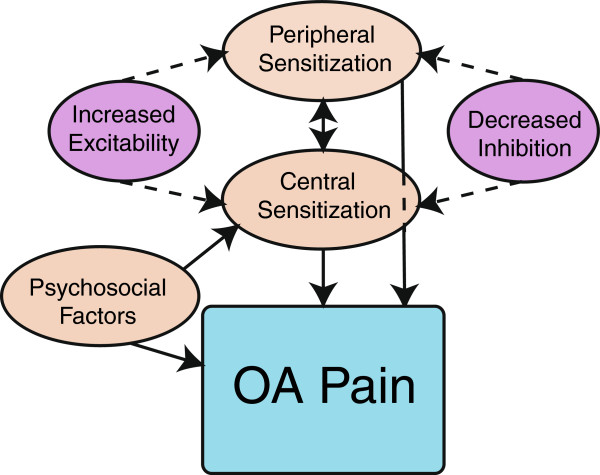
Schematic diagram of the interactions between neural (peripheral and central) and psychosocial factors in the modulation of osteoarthritic (OA) pain.

Several questions arise from this literature: Why do joints hurt? Do women have more pain than men? Do women have different underlying biological mechanisms that explain the differences in pain? What is the role of the nociceptor in the peripheral nervous system and what is the role of the central nervous system in generating and maintaining pain? What roles do psychosocial variables have in the OA pain and sex differences in OA pain? A schematic diagram representing potential mechanisms and modulating factors, that is, peripheral factors, central factors, and psychosocial factors that contribute to OA pain, is shown in Figure 1. This review examines the evidence for potential peripheral and central neural mechanisms responsible for the pain associated with knee OA, and how psychosocial variables influence the pain. We further identify gaps in research to further our understanding of sex differences in OA pain.

### Pain perception: an overview

The complexity of pain in OA is highlighted by the lack of correlation between individual pain and the degree of radiographic changes [3-5]. Even individuals with no history of knee pain can exhibit radiographic abnormalities consistent with early osteoarthritis such as osteophytes and joint space narrowing, or abnormalities on MRI such as cartilage damage, bone marrow lesions, osteophytes, synovitis or effusion [3-5]. Conversely, some individuals with knee pain present with no joint abnormalities [4,6]; no differences were observed between the sexes [6]. These inconsistent associations between joint abnormalities and pain, and the persistent pain that can continue after total knee replacement, suggest that factors other than nociceptor activation are involved in maintaining the pain. It is likely that central sensitization is responsible for the persistent and elevated pain experienced by individuals in whom the pain is disproportionately greater than the damage in the joint.

**Figure 2:**
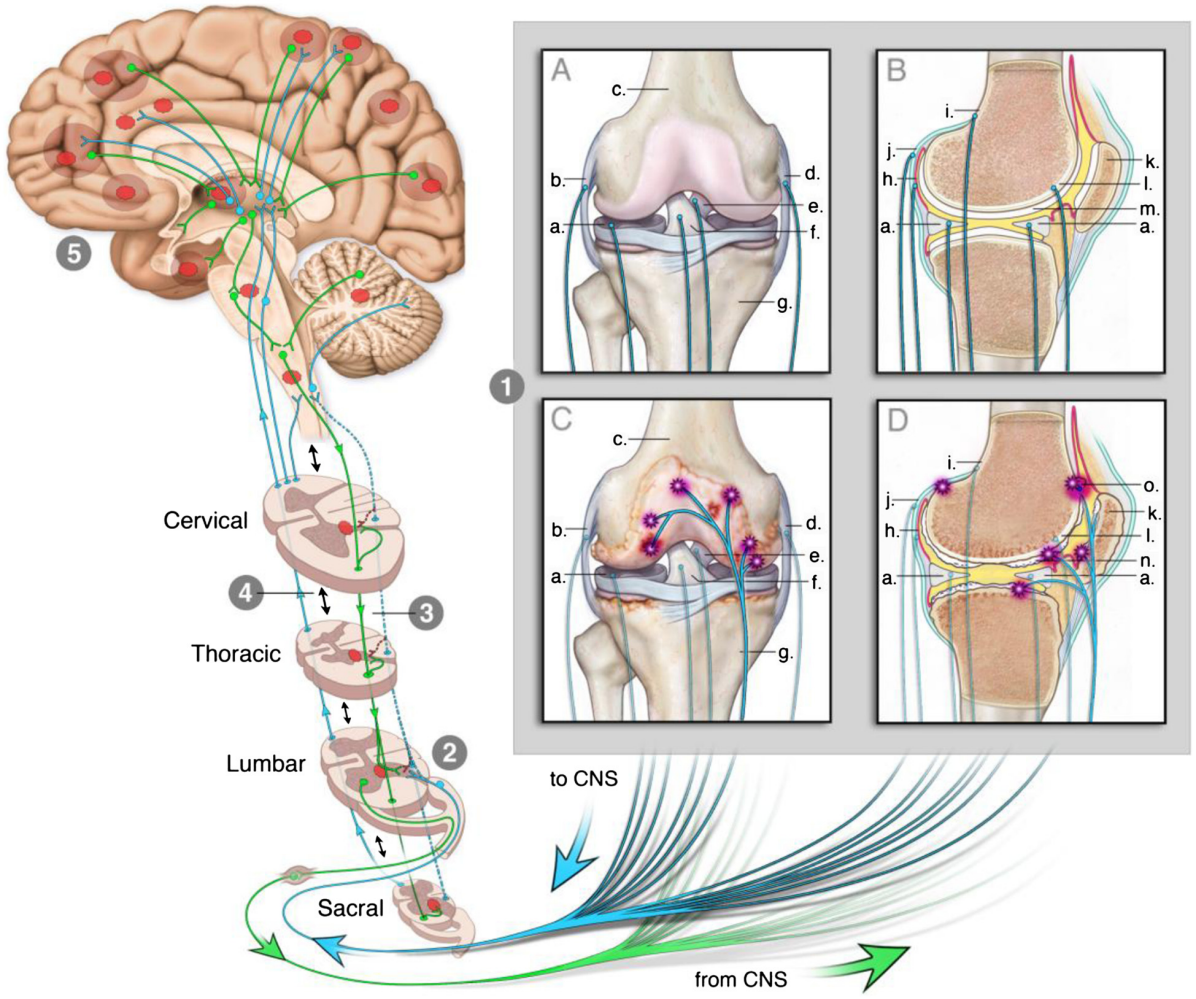
**This figure illustrates how the knee can engage the nervous system to give rise to different types of pain associated with knee OA. Part 1** shows anterior (1**A**, 1**C**) and lateral (1**B**, 1**D**) views of the healthy (1**A**, 1**B**) and OA knee (1**C**, 1**D**). Blue lines emerging from the knee depict nociceptors conveying information from the knee to the CNS (lumbar spinal cord). Green lines depict postganglionic sympathetic fibers sending efferent information from the CNS to the knee. Only sensory fibers are shown in 1a-d. In the OA knee, sensory and sympathetic fibers sprout branches into articular cartilage (* in 1**C**, 1**D**). **Part 2** illustrates the connection between the knee and the spinal cord. Sensitized afferent fibers can sensitize neurons in the lumbar dorsal horn. This ‘central sensitization’, shown by the red circle in the spinal cord, can become independent of and is modulated differently from ‘peripheral sensitization’. **Part 3:** Input from nociceptors to the spinal cord is concentrated in the segment associated with the body part the nociceptors innervate (lumbar segments). However, branches of nociceptors also extend to other rostral and caudal segments (blue lines) - normally, nociceptors have minimal impact on neurons in these segments. When nociceptors become sensitized they increase input to the spinal cord and sensitize central neurons in the innervated and uninnervated segments (green lines; red asterisks). **Part 4:** Multiple intersegmental excitatory and inhibitory spinal connections exist to coordinate nociceptive information (double-arrowed black lines). Central sensitization (red circles) is modulated by this inter-segmental communication. **Part 5:** Multiple connections exist that ascend from the spinal cord to the brain (blue lines) and descend from the brain to the spinal cord (green lines). Thus, input from the spinal cord engages neurons throughout the brain via complex ascending and descending systems. Input from sensitized spinal neurons can influence activity throughout the neuraxis altering normal processing of nociceptive and non-nociceptive information. Some regions that can be influenced are depicted by red circle. **a**, meniscus; **b**, lateral collateral ligament; **c**, distal femur; **d**, medial collateral ligament; **e**, posterior cruciate ligament; **f**, anterior cruciate ligament; **g**, proximal tibia; **h**, synovium; **i**, periosteum; **j**, joint capsule; **k**, patella; **l**, subchondral bone; **m**, normal articular cartilage; **n**, arthritic articular cartilage; **o**, osteophyte. Figure was adapted from [7].

Injury to a tissue is often accompanied by plastic changes in the nervous system that augment the excitability of neurons. Some nociceptive peripheral afferents, for example, increase spontaneous (background) activity and exhibit a greater responsiveness to noxious stimuli applied to the periphery – termed peripheral sensitization. Similarly, the properties of neurons throughout the CNS can also change by increasing spontaneous activity and responsiveness to peripheral stimuli – termed central sensitization. Figure 2 shows pathways in the peripheral and central nervous system that contribute to OA pain.

OA pain is associated with local pain and increased sensitivity to noxious stimulation of the involved joint (primary hyperalgesia). The local pain and primary hyperalgesia are likely due to peripheral sensitization. People with OA also have 1) pain beyond the involved limb (referred pain), 2) increased sensitivity to noxious stimuli beyond the site of injury (secondary hyperalgesia), and 3) an increased sensitivity to innocuous stimuli (allodynia). The referred pain, secondary hyperalgesia, and allodynia are probably a consequence of central sensitization.

People with pain, including those with OA, likely have both peripheral and central sensitization. In some cases, the pain is directly related to sensitization and activation of nociceptors in the damaged joint and correlates with the degree of joint effusion and synovial thickening [8,9]. Significantly decreasing nociceptor activity by performing joint replacement surgery provides the majority of patients with a relatively pain-free joint. In some patients, however, the pain does not match the degree of joint damage and continues even after total joint replacement [10]. It is important to recognize that following joint replacement, the capsule, ligamentous structures, and part of the synovium remain intact, and thus there is still nociceptor innervation of joint structures. The occurrence of persistent pain at 3–6 months after total knee replacement may be as high as 35% [10], but this percentage decreases to 13% at one year [11] and 10% at 7 years [12]. None of these studies compared the differences in pain after total knee replacement between men and women.

Predictive factors associated with the persistence of chronic pain after total knee replacement in patients with OA include high pain ratings prior to surgery, high pain ratings in the immediate postoperative period, multiple pain sites, loss of central inhibition preoperatively, higher pain catastrophizing, and the sex of the individual, with women at greater risk of chronic pain after surgery including those with total knee replacement or thoracotomy [2. 12]. Furthermore, the risk of developing chronic pain after total knee replacement can be correlated to preoperative symptoms of depression and anxiety, pain catastrophizing, as well as the presence of poor coping strategies [13-19].

Differences between men and women with osteoarthritis are complicated by the fact that women may also be pre- or post-menopausal. Post-menopausal women may also be undergoing replacement therapy. These factors are rarely considered in the analysis and determination of factors that contribute to differences in pain and function in osteoarthritis. One interesting study showed no difference in pain or pain thresholds in post-menopausal women receiving hormone replacement therapy (n=32), post-menopausal women not receiving hormone replacement therapy (n=42) and men (n=58) with osteoarthritis. However, men show a greater prevalence of osteoarthritis prior to age 45 and women show a greater prevalence after age 45 [20]. Together these data suggest future studies should more closely examine sex differences across the lifespan, as well as the influence of hormones on these sex differences.

In summary, multiple factors likely contribute to OA pain. Changes in nociceptor sensitivity may drive some of the pain of OA. The increased nociceptor activity can further drive changes in the central nervous system to augment and prolong pain independent of the nociceptor activity. Lastly, psychosocial variables can influence and modulate pain further contributing to the pain observed in people with OA. How these variables differ across the sexes, and the gaps in our knowledge related to sex differences, will be highlighted in the following sections focusing on 1) peripheral factors, 2) central factors, and 3) psychosocial factors that contribute to OA pain.

### Peripheral factors contributing to osteoarthritic pain

Nociceptors normally innervate the periosteum, meniscus, subchondral bone, synovium, capsule, and ligaments [21] (human, rat, cat, monkey) and thus information arising from any of these joint structures could contribute to the pain associated with OA. The exception is cartilage, which is aneural and avascular, although Schwab and Funk [22] found that branches of some periosteal nerve fibers penetrated the hyaline articular cartilage in close proximity with chondrocytes and near to the insertion of tendons and muscles (in rat). Cartilage degradation is a hallmark of OA, and denuded subchondral bone resulting from cartilage damage may be responsible for OA pain during joint movement due to the mechanical activation of nociceptors innervating the subchondral bone. Recent data, for example, show sprouting of nerve fibers to joint tissues that were previously not innervated, such as the presence of sympathetic and sensory nerves in vascular channels within the articular cartilage. Sprouting of nerve fibers also occurs in the subchondral bone and in osteophytes [23,24]. Thus, new nerve growth may result in enhanced activity of nociceptors from injured tissues. It is not yet known if there are differences in sprouting of nerve fibers between the sexes that could underlie the sex differences in pain.

Synovium is also highly innervated, and OA often involves synovial inflammation [21]. Increases in neuropeptide and ion channels expression in nociceptors innervating the synovium are observed in animal models associated with synovial inflammation, and in people with OA [21]. Inflammatory mediators, such as prostaglandins and pro-inflammatory cytokines (e.g., IL-6, IL-1beta, TNF) activate and sensitize nociceptors [25], which is manifested as increased spontaneous activity of the nociceptor and increased response to mechanical stimulation associated with joint movement [21,25].

Few data are available on the influence of sex hormones on innervation patterns or nociceptor activity, particularly as these factors relate to OA. However, two studies of the temporomandibular joint (TMJ) suggest augmented excitability of nociceptors in association with estrogens. Estradiol increases the excitability of nociceptors innervating the TMJ as evidenced by a decrease in action potential threshold and an increase in the incidence of spontaneous activity in ovariectomized rats with estradiol replacement compared with ovariectomized rats without replacement [26]. Further, nociceptors innervating the TMJ of female rats show a greater response to intraarticular injection of glutamate compared with those from male rats [27]. These data suggest that nociceptors in females are more excitable and exhibit augmented activity in response to noxious stimuli. It is unknown at this point if there are changes in nociceptor excitability after menopause when circulating estradiol would decrease, or if these changes persist due to local sources of estradiol in joint structures (see accompanying review by Hart et al. entitled “Hormonal modulation of connective tissue homeostasis and sex differences in risk for osteoarthritis of the knee”).

It has also been suggested that opioid agonists can be less potent and efficacious in females than males [28]; however, the data may depend on species, disease process, and age [29,30]. For example, opioids appear to be less efficacious in female rodents than males, whereas opioids seem to be more efficacious in women than men [30,31]. A recent review of the literature suggests that μ- and μ/κ-mixed agonists are more effective in women, particularly for morphine [32]. However, most studies examine postoperative or emergency-room pain, morphine, and short-duration usage. Meta-analysis of trials of efficacy in people with osteoarthritis show a significant reduction in pain and improved physical function with moderate effect sizes [33]. Although opioids have been shown to be efficacious in people with osteoarthritis, it is unclear if there are sex differences in opioid efficacy in this population or with more long-term usage. After injury, including OA, there is an upregulation of endogenous opioid peptides in inflammatory cells at the site of insult as well as an up-regulation of opioid receptors on peripheral terminals of nociceptors innervating the injured tissue [34]. In individuals with OA, this up-regulation of opioid receptors and peptides increases endogenous inhibition locally because intraarticular blockade of opioid receptors with naloxone increases the arthritic pain [34]. One possibility is that these changes in opioids may be explained by the influence of sex hormones on the peripheral expression and potency of endogenous opioid peptides and their receptors. Thus, the changes in endogenous inhibition, particularly the opioid pathway, may contribute to the greater pain observed in women with OA than in men.

In summary, peripheral factors can contribute to pain in knee OA (Table 2); however, little is known about sex differences in these peripheral factors (Table 3). The growth of new nociceptors, activation of nociceptors in the subchondral bone exposed after cartilage degradation, and nociceptors innervating synovium sensitized by inflammatory mediators could all augment the peripheral input to the central nervous system and result in pain. Increased nociceptor activation by noxious stimuli and reduced inhibition in the periphery could also contribute to the greater pain observed in women with OA.

**Table 2:** Peripheral mechanisms of knee OA

**Study**	**Male**	**Female**	**Not stated**	**Findings**
Cairns et al., 2001 [27]	Rat	Rat		Glutamate injected into masseter muscle produced greater nociceptor excitation in female rats.
Cook and Nickerson, 2005 [31]	Rat	Rat		Mu-opioid agonists are more effective in male vs. female arthritic rats; effects are both peripheral and central.
Flake et al., 2005 [26]		Rat		Compared ovariectomized with and without estrogen; Estrogen increases nociceptor excitability of TMJ neurons in cells from uninjured and inflamed rats.
Heppleman, 1997 [21]			Rat, cat, dog, monkey	Review discussing innervation of joint structures including 71 references. No mention of male or female in text.
Schaible et al., 2010 [25]			Rats, mice	Review discussing effects of cytokines on joint afferents including 55 references. No mention of male or female in text.
Schwab and Funk, 1998 [22]			Rat	Shows neuropeptide innervation of hyaline cartilage and fibrocartilage.
Stein, 1995 [34]			Rat; human; cultured cells	Review article discussing peripheral opioid effects in arthritis including 50 references; no mention of sex differences.
Suri et al., 2007 [23]	Human	Human		Shows innervation of articular cartilage in subjects with OA; no mention of sex differences.
Walsh et al., 2010 [24]	Human	Human		Shows vascularization and innervation of RA and OA joints. No mention of sex differences.

**Table 3:** Identified gaps in sex/gender differences in knee OA

**Gaps related to peripheral nervous system**	
Gap 1	Are there sex differences in nociceptor innervation of the OA joint?
Gap 2	Do sex hormones modify knee joint nociceptor activity?
Gap 3	Do nociceptors innervating the OA joint respond differently to inflammatory stimuli between the sexes?
Gap 4	Does peripheral opioid peptide and receptor upregulation after knee OA differ between the sexes?
Gaps related to the central nervous system	
Gap 5	Are there sex differences in processing of nociceptive information from knee in the central nervous system in the healthy or diseased knee?
Gap 6	Are there sex differences in brain activation patterns associated with knee OA pain?
Gap 7	Do sex hormones modulate central neuronal activity associated with nociception in the healthy or diseased knee?
Gap 8	Are there sex differences in OA patients for measures of temporal summation (central excitability) and conditioned-pain modulation (central inhibition)?
Gaps related to psychosocial factors	
Gap 9	Are there sex differences in psychosocial variables in OA such as depression, anxiety, self-efficacy, pain catastrophizing, and fear of pain?
Gaps that encompass all factors	
Gap 10	Are different pharmacologic and non-pharmacologic strategies needed to address treatment of adverse psychosocial variables between sexes?
Gap 11	Might effective pain management strategies to reduce pain in OA differ between women and men?
Gap 12	What is the influence of age on sex differences?

### Pain is created by the central nervous system

**Figure 3:**
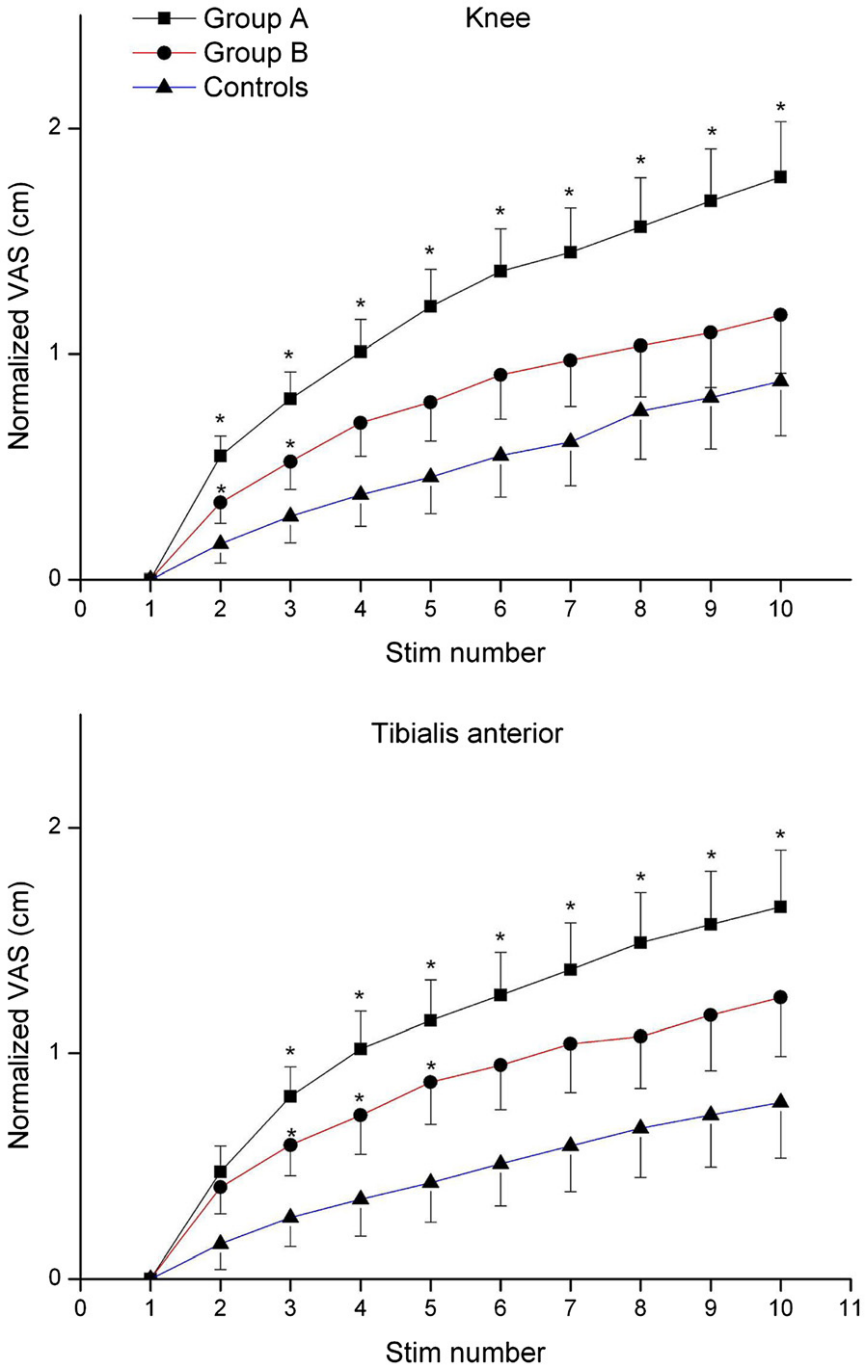
**Example of temporal summation in response to pressure applied either to the knee joint or to the anterior tibialis muscle at the pressure-pain threshold in people with osteoarthritis.** VAS scores were normalized for comparison by subtracting the first stimulus pain score from subsequent pain scores. Group A had more clinical pain with VAS scores of 6/10 or greater and Group B had clinical pain scored less than 6/10. These two groups were compared to age and sex-matched controls. The greatest temporal summation occurred in the group with the highest pain scores. *, p<0.05 Group A different from controls and Group B; Group B different from Controls. Reprinted from [5] with permission of the International Association for the Study of Pain.

The CNS creates and modulates the experience of pain whether derived and maintained from nociceptive input (e.g. knee arthritis) or maintained independent of nociceptive input. Pain is a product of a dynamic balance between facilitation and inhibition of information processing. In people with chronic OA, there is increased central excitability and decreased central inhibition [5]. Central excitability can be assessed by testing “temporal summation,” which corresponds to a progressive increase in reported pain in response to the same noxious stimulus given repetitively. People with OA show augmented temporal summation to noxious stimulation compared with controls, and temporal summation among people with osteoarthritis is greater in those with higher pain than in those with lower pain (> 6 VAS vs. <6 VAS) [5] (Figure 3). In addition, temporal summation significantly correlates with other aspects of OA pain, such as pain duration and pain during walking, suggesting central excitability may underlie these pain measures as well [5]. Furthermore, infusion of hypertonic saline into the tibialis anterior muscle in people with knee OA results in greater areas of referred pain and longer lasting sensitivity to noxious stimuli [35], and there are widespread increases in sensitivity to noxious stimuli (secondary hyperalgesia) among people with OA [36].

Conditioned pain modulation, also referred to as diffuse noxious inhibitory controls (DNIC), is based on the finding that some neurons in the spinal cord of rodents are inhibited when a noxious stimulus is applied beyond the excitatory receptive field of the neuron [37]. This observation presumably underlies the reduction in pain experienced by humans in one area of the body when a noxious stimulus is applied to another area. This type of inhibitory control is reduced in people with OA [5,36], but the depression of conditioned pain modulation returns to normal after total joint replacement in patients who are pain-free [36]. It is unclear if those who have pain after TKA have reduced conditioned pain modulation, as occurs in other postoperative conditions [38]. Thus, people with OA have both altered central excitability and altered central inhibition (Figure 1).

Central processing appears to differ between the sexes. For example, there is greater temporal summation to both heat and mechanical stimuli in healthy women compared with men [39], suggesting enhanced central excitability. Healthy men show a greater decrease in temporal summation and increase in pain thresholds in response to conditioned pain stimulation when compared with women [40,41], suggesting that women have less effective central inhibition than men. Together, these data indicate that the normal state for women is a greater ability to produce central sensitization to peripheral stimuli and less effective inhibitory systems, but there is evidence that central inhibitory mechanisms vary with the menstrual cycle, which may temper these conclusions [42]. Overall, little is known about sex differences in central excitability and central inhibition in people with OA.

Brain imaging studies have shown that multiple cortical and subcortical sites in the brain are engaged when an individual experiences pain. When data are collapsed across subjects, the sites whose components are activated most consistently are referred to as the “pain matrix,” although there are individual differences and areas outside this matrix can be involved (e.g., cerebellum). Classical somatosensory regions, including the somatosensory cortex, thalamus, and spinal cord, that are activated by noxious stimuli likely mediate the sensory component of nociception (i.e., recognition of injury). In addition, the anterior cingulate cortex, insular cortex, amygdala, and prefrontal cortex are also activated when an individual experiences pain. These regions may contribute to the emotional aspect of the pain experience, including unpleasantness and fear as well as the associated anxiety and depression.

In patients with OA, painful mechanical knee stimulation is associated with enhanced brain activity in the thalamus, somatosensory cortex, cingulate cortex, and amygdala [43,44]. Brain activity decreases after the application of intra-articular lidocaine to the osteoarthritic knee, and changes in the thalamus correlate with changes in pain ratings [43]. When patients with OA are compared with healthy controls, spontaneous pain of OA activates the prefrontal limbic regions, and this pain and activity is reduced by a cyclooxygenatase-2 inhibitor [45], suggesting these regions are critical for spontaneous pain. Furthermore, there is a reduction in gray matter volume of the thalamus in people with hip OA that is reversed at 9 months after total hip replacement [46]; however the clinical significance of reduced gray matter volume is unclear. The activation of the pain matrix and the reduction in gray volume have been observed in multiple pain conditions [47]. Thus, OA-associated changes in components of the pain matrix and perhaps other regions are modifiable with effective treatments.

Subcortical sites activated during pain include areas in the brainstem, such as the periaqueductal gray (PAG) and the rostral ventromedial medulla (RVM). A key connective pathway appears be from the PAG to the RVM and thence to the spinal cord to inhibit activity of nociceptive spinal neurons and thereby reduce central sensitization; this pathway is critical in opioid analgesia. The PAG-RVM-spinal cord pathway contains endogenous opioid peptides and receptors, and when activated reduces central sensitization and hyperalgesia through activation of opioid peptides. Neurons in this pathway can also elicit the converse modulation and increase excitability of nociceptors to enhance pain, thereby providing a supraspinal mechanism for pain facilitation. Brain imaging studies in people with OA show greater activation of this facilitatory pathway in response to noxious stimulation in referred pain areas [48], a measure of secondary hyperalgesia. Because activity in these brainstem sites correlates with measures of centrally-mediated pain symptoms in people with OA [48], subcortical activation in the PAG and RVM may predict central sensitization. Accordingly, hyperalgesia is reversed by blockade of neural activity in the RVM or the PAG in animal models of musculoskeletal pain [49].

Sex differences in these subcortical brain sites may help explain differences in opioid analgesic potency and efficacy between male and female rodents [28]. For example, male rats express more mu-opioid receptors in the PAG that correlate positively with the difference in morphine analgesia [28] supporting the fact that that males require less morphine than females. Female rats have a greater number of neurons that project from the PAG to the RVM and fewer of these neurons are activated by tissue injury [28], suggesting that females do not activate inhibitory pathways to the same extent as males. These data indicate that subcortical brainstem sites are key modulators of nociception and that differential expression of sex-steroid receptors may alter nociceptive processing.

In this context, sex differences in opioid analgesia are a topic of considerable interest both experimentally and clinically. Initial findings led to the conclusion that whereas opioid analgesia was greater in female rodents than males, the opposite was the case in humans reviewed in [50]. Since then, however, it has become clear that the situation is not straightforward; conclusions seem premature because so many different genetic, physiological, hormonal, pharmacologic, and psychosocial factors contribute to sex differences in opioid analgesia in both animal models and humans [30,51-54].

Within the spinal cord, sex differences also occur in the processing of nociceptive information. As an example, male mice that do not express a functional Toll-like receptor 4 (TLR4) do not develop pain-behaviors after either nerve injury or inflammation, blockade of TLR4 in the spinal cord reduces pain- behaviors in mice, and activation of TLR4 in the spinal cord produce pain-behaviors. These effects do not occur in female mice as they still exhibit pain behaviors without a functional TLR4 receptor, TLR4 antagonists have no effect on pain-behaviors, and TLR4 activation has no effect [55]. The TLR4 effect on pain behaviors in males is dependent on testosterone [55]. Moreover, females exhibit greater sensitivity of nociceptive dorsal horn neurons to noxious visceral stimulation than males that depends on estrogen and is associated with greater spinal expression of excitatory neurotransmitter receptors (GluN1) [56,57]. Also, morphine acts via μ- and κ-opioid receptors in the spinal cord of females but only via μ-opioid receptors in males [51,58]. This sex difference is due to a difference in the molecular structure of opioid receptors in the spinal cord showing a greater prevalence of a mu-opioid and kappa-opioid receptor heterodimers that changes across the estrous cycle [51]. Thus, structural and molecular changes can differ between the sexes that may explain some of the underlying differences in pain sensitivity. However, how these relate to the nociceptive transmission from the joint or in osteoarthritis is unknown.

In summary, central nervous system factors contribute to pain in knee OA (Table 4). The enhanced central excitability and reduced central inhibition in women could lead to prolonged and greater pain that does not directly match the degree of injury. Differences in processing of nociceptive information in supraspinal brain sites could explain the differences observed between men and women with osteoarthritis; however, these studies have yet to be done (Table 3). Despite the data on sex differences in processing of painful stimuli at multiple central nervous system sites [59,60], there are virtually no data on sex differences that occur after stimulation of joint tissue, after injury to a joint, or in osteoarthritis.

**Table 4:** Central mechanisms of knee OA

**Study**	**Male**	**Female**	**Findings**
Arendt-Nielsen et al., 2010 [5]	Human	Human	Increased central excitability in OA subjects; greater excitability with greater OA pain; lack of correlation between radiographic findings and pain; no discussion of sex differences.
Arendt-Nielsen et al., 2008 [41]	Human	Human	Healthy men show greater decrease in central excitability in response to conditioned pain modulation.
Bajaj et al., 2001 [35]	Human	Human	Hypertonic saline infusion into tibialis anterior shows greater areas of referred pain; no discussion of sex differences.
Baliki et al., 2008 [43]	Human	Human	fMRI shows enhanced activity in thalamus, somatosensory cortex, cingluate cortex, and amygdala in OA patients in response to painful mechanical stimulation; decreased brain activity in response to intra-articular lidocaine.
Ge et al., 2005 [61]	Human	Human	Reduced pain ratings to a second intramuscular injection of glutamate in men compared to women.
Bwilym et al., 2010 [46]	Human	Human	Imaging study shows that atrophy of thalamus in hip OA is reversed by total hip replacement; no sex differences reported.
Gwilym et al., 2009 [48]	Human	Human	Imaging study shows patients with OA have increased activity in brainstem facilitation pathway that is correlated with neuropathic symptoms; no sex differences reported.
Kulkarni et al., 2007 [44]	Human	Human	Reduction in conditioned pain modulation in OA, that is reversed after total joint replacement in pain-free individuals; no sex differences reported.
LeBars et al., 1979 [37]	Rat		Activation of diffuse noxious inhibitory control pathways reduces activity of nociceptive neurons in spinal cord.
Loyd and Murphy, 2006 [62]	Rat	Rat	Review article including 141 references describing sex differences in central pain modulation.
Parks et al., 2011 [45]	Human	Human	fMRI in knee OA shows spontaneous pain activates prefrontal-limbic regions; COX-2 inhibitor decreases spontaneous pain and activity in prefrontal-limbic regions; no sex differences reported.
Sarlani and Greenspan, 2002 [39]	Human	Human	Greater temporal summation to heat and mechanical stimuli in healthy women than men.
Staud et al., 2003 [40]	Human	Human	Conditioned pain modulation is more effective in heathy men than women.
Tousignant-Laflamme and Marchand, 2009 [42]		Human	Menstrual cycle alters conditioned pain modulation in healthy women.
Yarnitsky et al., 2008 [38]	Human	Human	Lower conditioned pain modulation prior to surgery is predictive of postoperative chronic pain.

### Psychosocial factors that influence pain

Pain is a multidimensional experience that includes psychological, emotional, environmental, and social factors. Literature on environmental and social factors related to sex differences in OA pain is limited, although there is a growing literature on social factors related to knee OA surgery [15,63,64]. This section of the review focuses on psychological and emotional factors. There is a wide variation in how people cope with pain, how they think, interpret and report pain, as well as psychosocial and environmental factors that can influence pain. These constructs can influence the experience of pain in both positive and negative ways [65]. For example, pessimistic patients have more moderate-to-severe pain than non-pessimistic patients two years after total knee replacement surgery [2]. Individuals with chronic pain use a mixture of active coping strategies (distraction, modifying activity) as well as passive coping strategies (resting, ignoring the pain). Those who rely on passive strategies are more likely to have greater pain and reduced function [66]. Environmental factors, such as social support can influence the timing and recovery from total knee arthroplasty [63], but does not appear to differ between the sexes [15]. It is possible that some of these coping strategies and psychosocial factors can differ between men and women. Through education and exercise, coping strategies can become active and thereby improve psychosocial factors such as depression, anxiety, pain catastrophizing, self-efficacy, and fatigue.

The way an individual thinks about and interprets pain explains some of the variability in pain among individuals, and is particularly relevant to sex differences in pain [67]. Men tend to be more stoic, under-report pain, and endure more pain because of social and cultural norms. In contrast, women are more alert to the potential danger of injury, tend to experience pain with greater emotion, worry more about the consequences of pain, seek more treatment for it, and have higher use of prescription and over-the-counter medications [67]. Women tend to use a greater repertoire of coping strategies, such as seeking social support, relaxation and distraction, and active behavioral and cognitive coping mechanisms [67]. In contrast, men tend to rely more on more focused approaches, such as problem solving, denial, optimism, and tension-reducing activities, such as alcohol consumption [67]. Understanding the differences in the way men and women process, discuss, and cope with pain can inform effective treatment programs.

Two constructs that have emerged as strong predictors of pain and therapeutic outcomes in a variety of pain conditions are pain catastrophizing and self-efficacy. Pain catastrophizing reflects the tendency to focus and magnify the consequences of the pain and to feel helpless in managing the pain. Self-efficacy is the belief that one can achieve a desirable outcome, such as pain relief or improve activities of daily living. Higher catastrophizing scores are associated with greater pain and disability 6 weeks after total knee replacement [14], poor outcome 6 months after total knee replacement [68], and contribute to reduced function in late stage knee OA [15]. However, higher pain catastrophizing was not related to pain during movement or physical activity levels in a sample of 208 people with late-stage knee OA [15].

Higher self-efficacy scores in pain and functional activities directly correlate with less pain and greater function, respectively [69]. Further, self-efficacy beliefs for pain underlie the relation between pain catastrophizing and pain [70]. Therapeutic strategies that increase self-efficacy can reduce catastrophizing and improve pain and function. For example, self-management strategies (such as exercise and activity management) can improve self-efficacy and pain catastrophizing, and have been shown to reduce pain and improve function in people with OA [19]. Similarly, behavioral interventions just prior to surgery improved self-efficacy, decreased pain, and improved function [16]. A recent study showed no difference between men and women in pain catastrophizing in people with late stage OA [15]. Although catastrophizing can influence such sex differences in pain-related outcomes as pain intensity and disability, it is unknown whether women undergoing TKA are more likely to catastrophize than men.

Another construct that is highly associated with pain and function in OA is fear of pain. Fear of pain refers to an excessive expectation that physical activity will worsen pain and function. People with pain-related fear are likely to avoid physical activity, which decreases function and increases disability. Maintaining physical activity and exercise are critical self-management skills that are effective for reducing pain and improving function in people with OA [71]. Pain-related fear explains significant amounts of variance in physical disability (25%), pain (13%), and function (1%) [72]. At present, it is not known whether pain-related fear contributes to sex differences in OA pain and function.

Depression and anxiety can also influence pain associated with OA. People with OA have a higher incidence of depression and anxiety than the general population and are associated with greater pain intensity, worse symptoms, greater healthcare utilization, and report more pain after total joint replacement [17]. In addition, people with depression have lower functional scores and slower recovery after total joint replacement [18]. One study found that depression scores in Chinese elders with OA explained a portion of the sex differences in pain [73], but the general pain literature does not support the conclusion that depression is an explanatory factor [74]. However, how anxiety and depression contribute to sex differences in pain and function among people with OA is unknown.

In summary, psychosocial variables contribute to pain variability (Table 5). No single factor is likely to explain all of the sex differences in pain [20], including pain experienced by individuals with OA. Although some investigators have begun to examine the interactions among multiple psychosocial variables in people with OA, there are few such data particularly related to sex differences (Table 3). Pain catastrophizing and self-efficacy contribute to the pain and functional deficits associated with OA, but little is known about their interaction with such factors as depression and anxiety. Similarly, little is known about the influence of environmental and social factors. Treatment strategies based on the psychosocial profile of an individual and possible sex differences need to be developed to improve pain outcomes.

**Table 5:** Psychosocial variables in knee OA

**Study**	**Findings**
Dekker et al., 2009 [17]	People with OA have a higher prevalence of depression and anxiety and this is associated with worse pain and greater healthcare utilization; sex differences not reported.
Fransen et al., 2002 [71]	Systematic review showing exercise reduces pain and improves function in OA; sex differences not reported.
Lamb et al., 2008 [16]	Behavioral interventions prior to surgery improve self-efficacy, decrease pain, and improve function in OA; sex differences not reported.
Lorig et al., 2008 [19]	Self-management strategies improve self-efficacy and pain catastrophizing in OA; sex differences not reported.
Marks et al., 2009 [18]	People with depression have reduced function and recover slower after total joint replacement; sex differences not reported.
Pells et al., 2008 [69]	Higher self-efficacy scores in pain and function correlate with lower pain and greater function in OA; sex differences not reported.
Perrot et al., 2008 [66]	Passive coping strategies generally result in higher pain and lower function; sex differences not reported.
Riddle et al., 2010 [68]	Higher catastrophizing scores are associated with poor outcome 6 months after total knee replacement; sex differences not reported.
Shelby et al., 2008 [70]	Self-efficacy beliefs underlie the relation between pain catastrophizing and pain; sex differences not reported.
Singh et al., 2008 [2]	Pessimistic patients have more moderate-to-severe pain 2 years after total knee replacement; sex differences not reported.
Somers et al., 2009 [72]	Pain-related fear explains part of the variance in physical disability, pain, and function in OA; sex differences not reported.
Sullivan et al., 2009 [14]	Higher pain catastrophizing scores are associated with greater pain and disability 6 weeks after total joint replacement; sex differences not reported.
Tonelli et al., 2011 [15]	Shows greater pain during movement in women with late stage OA; no difference in depression, anxiety or pain catastrophizing between sexes in OA; models predictors of movement pain in women and men with OA.
Tsai, 2007 [73]	Depression tendency in OA explain a portion of the sex differences in pain.
Unruh, 1996 [67]	Review discussing sex differences in the clinical pain experience.

All studies were performed in human subjects and included both men and women.

## Conclusion

Pain in knee OA involves the peripheral and central nervous system whose processing is influenced by psychosocial variables. Sex differences exist, but our understanding of them is rudimentary. Persistent pain that continues after knee replacement surgery has healed occurs more in women than men. We have identified gaps in the literature as they relate to sex differences in OA pain (Table 3). These gaps focus on understanding sex differences in common factors thought to be associated with OA pain, including nociceptor innervation and sensitization, central nociceptive processing and sensitization, effects of sex hormones on neuron activation, and differences in psychosocial variables and coping strategies. One of the major gaps in the literature on osteoarthritis and pain is the differences between pre- and post-menopausal women. Research to improve understanding of chronic pain and the influence of sex and gender issues is essential to develop effective interventions to decrease pain and improve quality of life.

## Abbreviations

CNS: Central nervous system;DNIC: Diffuse noxious inhibitory controls;fMRI: Functional magnetic resonance imaging;IL: Interleukin;MRI: Magnetic resonance imaging;OA: Osteoarthritis;PAG: Periaqueductal gray;TRA: Rheumatoid arthritis;RVM: Rostral ventromedial medulla;TKA: Total knee arthroplasty;TMJ: Temporomandibular joint;TNF: Tumor necrosis factor;VAS: Visual analog scale

## Competing interests

The authors declare that they have no competing interests.

## Authors’ contributions

All authors contributed to the development of the review.

## References

[B1] O'Connor MI Implant survival, knee function, and pain relief after TKA. Are there differences between men and women? Clin Orthop Relat Res 2011 469 1846 1851 10.1007/s11999-011-1782-5 21267799 PMC3111790

[B2] Singh JA Gabriel S Lewallen D The impact of gender, age, and preoperative pain severity on pain after TKA Clin Orthop Relat Res 2008 466 2717 2723 10.1007/s11999-008-0399-9 18679762 PMC2565033

[B3] Beattie KA Boulos P Pui M O'Neill J Inglis D Webber CE Adachi JD Abnormalities identified in the knees of asymptomatic volunteers using peripheral magnetic resonance imaging Osteoarthritis Cartilage 2005 13 181 186 10.1016/j.joca.2004.11.001 15727883

[B4] Lethbridge-Cejku M Scott WW Jr Reichle R Ettinger WH Zonderman A Costa P Plato CC Tobin JD Hochberg MC Association of radiographic features of osteoarthritis of the knee with knee pain: data from the Baltimore Longitudinal Study of Aging Arthritis Care Res 1995 8 182 188 10.1002/art.1790080311 7654803

[B5] Arendt-Nielsen L Nie H Laursen MB Laursen BS Madeleine P Simonsen OH Graven-Nielsen T Sensitization in patients with painful knee osteoarthritis Pain 2010 149 573 581 10.1016/j.pain.2010.04.003 20418016

[B6] Stehling C Liebl H Krug R Lane NE Nevitt MC Lynch J McCulloch CE Link TM Patellar cartilage: T2 values and morphologic abnormalities at 3.0-T MR imaging in relation to physical activity in asymptomatic subjects from the osteoarthritis initiative Radiology 2010 254 509 520 10.1148/radiol.09090596 20019141 PMC2809928

[B7] Stratton P Berkley KJ Chronic pelvic pain and endometriosis: translational evidence of the relationship and implications Hum Reprod Update 2011 17 327 346 10.1093/humupd/dmq050 21106492 PMC3072022

[B8] Fei AI Yu C Zhang W Morelli JN Xiaoming L MR imaging of knee osteoarthritis and correlation of findings with reported patient pain J Huazhong Univ Sci Technol 2010 30 248 254 10.1007/s11596-010-0223-0 20407883

[B9] Hill CL Gale DG Chaisson CE Skinner K Kazis L Gale ME Felson DT Knee effusions, popliteal cysts, and synovial thickening: association with knee pain in osteoarthritis J Rheumatol 2001 28 1330 1337 11409127

[B10] Puolakka PA Rorarius MG Roviola M Puolakka TJ Nordhausen K Lindgren L Persistent pain following knee arthroplasty Eur J Anaesthesiol 2010 27 455 460 10.1097/EJA.0b013e328335b31c 20299989

[B11] Brander VA Stulberg SD Adams AD Harden RN Bruehl S Stanos SP Houle T Predicting total knee replacement pain: a prospective, observational study Clin Orthop Relat Res 2003 416 27 36 14646737 10.1097/01.blo.0000092983.12414.e9

[B12] Garcia JA Bewley B Redden JF The St. Leger total knee replacement-a 7-year clinical assessment and survivorship analysis Knee 2003 10 173 177 10.1016/S0968-0160(02)00096-0 12788002

[B13] Forsythe ME Dunbar MJ Hennigar AW Sullivan MJ Gross M Prospective relation between catastrophizing and residual pain following knee arthroplasty: two-year follow-up Pain Res Manag 2008 13 335 341 18719716 10.1155/2008/730951 PMC2671320

[B14] Sullivan M Tanzer M Stanish W Fallaha M Keefe FJ Simmonds M Dunbar M Psychological determinants of problematic outcomes following Total Knee Arthroplasty Pain 2009 143 123 129 10.1016/j.pain.2009.02.011 19304392

[B15] Tonelli SM Rakel BA Cooper NA Angstom WL Sluka KA Women with knee osteoarthritis have more pain and poorer function than men, but similar physical activity prior to total knee replacement Biol Sex Differ 2011 2 12 10.1186/2042-6410-2-12 22074728 PMC3228720

[B16] Lamb SE Toye F Barker KL Chronic disease management programme in people with severe knee osteoarthritis: efficacy and moderators of response Clin Rehabil 2008 22 169 178 10.1177/0269215507080764 18212037

[B17] Dekker J van Dijk GM Veenhof C Risk factors for functional decline in osteoarthritis of the hip or knee Curr Opin Rheumatol 2009 21 520 524 10.1097/BOR.0b013e32832e6eaa 19550331

[B18] Marks R Comorbid depression and anxiety impact hip osteoarthritis disability Disabil Health J 2009 2 27 35 10.1016/j.dhjo.2008.10.001 21122740

[B19] Lorig KR Ritter PL Laurent DD Plant K The internet-based arthritis self-management program: a one-year randomized trial for patients with arthritis or fibromyalgia Arthritis Rheum 2008 59 1009 1017 10.1002/art.23817 18576310

[B20] Berkley KJ Sex differences in pain Behav Brain Sci 1997 20 371 380 10097000 10.1017/s0140525x97221485

[B21] Heppelmann B Anatomy and histology of joint innervation J Peripher Nerv Syst 1997 2 5 16 10975732

[B22] Schwab W Funk RH Innervation pattern of different cartilaginous tissues in the rat Acta Anat (Basel) 1998 163 184 190 10.1159/000046497 10072566

[B23] Suri S Gill SE Massena CS de Wilson D McWilliams DF Walsh DA Neurovascular invasion at the osteochondral junction and in osteophytes in osteoarthritis Ann Rheum Dis 2007 66 1423 1428 10.1136/ard.2006.063354 17446239 PMC2111605

[B24] Walsh DA McWilliams DF Turley MJ Dixon MR Franses RE Mapp PI Wilson D Angiogenesis and nerve growth factor at the osteochondral junction in rheumatoid arthritis and osteoarthritis Rheumatology (Oxford) 2010 49 1852 1861 10.1093/rheumatology/keq188 20581375 PMC2936950

[B25] Schaible HG Von Banchet GS Boettger MK Brauer R Gajda M Richter F Hensellek S Brenn D Natura G The role of proinflammatory cytokines in the generation and maintenance of joint pain Ann N Y Acad Sci 2010 1193 60 69 10.1111/j.1749-6632.2009.05301.x 20398009

[B26] Flake NM Bonebreak DB Gold MS Estrogen and inflammation increase the excitability of rat temporomandibular joint afferent neurons J Neurophysiol 2005 93 1585 1597 15525813 10.1152/jn.00269.2004 PMC2838234

[B27] Cairns BE Sessle BJ Hu JW Characteristics of glutamate-evoked temporomandibular joint afferent activity in the rat J Neurophysiol 2001 85 2446 2454 11387390 10.1152/jn.2001.85.6.2446

[B28] Loyd DR Murphy AZ The role of the periaqueductal gray in the modulation of pain in males and females: are the anatomy and physiology really that different? Neural Plast 2009 2009 462879 19197373 10.1155/2009/462879 PMC2633449

[B29] Craft RM Mogil JS Aloisi AM Sex differences in pain and analgesia: the role of gonadal hormones Eur J Pain 2004 8 397 411 10.1016/j.ejpain.2004.01.003 15324772

[B30] Craft RM Sex differences in opioid analgesia: "from mouse to man" Clin J Pain 2003 19 175 186 10.1097/00002508-200305000-00005 12792556

[B31] Cook CD Nickerson MD Nociceptive sensitivity and opioid antinociception and antihyperalgesia in Freund's adjuvant-induced arthritic male and female rats J Pharmacol Exp Ther 2005 313 449 459 15608071 10.1124/jpet.104.077792

[B32] Niesters M Dahan A Kest B Zacny J Stijnen T Aarts L Sarton E Do sex differences exist in opioid analgesia? A systematic review and meta-analysis of human experimental and clinical studies Pain 2010 151 61 68 10.1016/j.pain.2010.06.012 20692097

[B33] Avouac J Gossec L Dougados M Efficacy and safety of opioids for osteoarthritis: a meta-analysis of randomized controlled trials Osteoarthritis Cartilage 2007 15 957 965 10.1016/j.joca.2007.02.006 17398122

[B34] Stein C The control of pain in peripheral tissue by opioids Mechanisms of Disease 1995 332 1685 1690 10.1056/NEJM199506223322506 7760870

[B35] Bajaj P Bajaj P Graven-Nielsen T Arendt-Nielsen L Osteoarthritis and its association with muscle hyperalgesia: an experimental controlled study Pain 2001 93 107 114 10.1016/S0304-3959(01)00300-1 11427321

[B36] Kosek E Ordeberg G Lack of pressure pain modulation by heterotopic noxious conditioning stimulation in patients with painful osteoarthritis before, but not following, surgical pain relief Pain 2000 88 69 78 10.1016/S0304-3959(00)00310-9 11098101

[B37] LeBars D Dickenson AH Besson J-M Diffuse noxious inhibitory controls (DNIC). II. Lack of effect on non-convergent neurones, supraspinal involvement and theoretical implications Pain 1979 6 305 327 10.1016/0304-3959(79)90050-2 460936

[B38] Yarnitsky D Crispel Y Eisenberg E Granovsky Y Ben-Nun A Sprecher E Best LA Granot M Prediction of chronic post-operative pain: pre-operative DNIC testing identifies patients at risk Pain 2008 138 22 28 10.1016/j.pain.2007.10.033 18079062

[B39] Sarlani E Greenspan JD Gender differences in temporal summation of mechanically evoked pain Pain 2002 97 163 169 10.1016/S0304-3959(02)00015-5 12031789

[B40] Staud R Robinson ME Vierck CJ Price DD Diffuse noxious inhibitory controls (DNIC) attenuate temporal summation of second pain in normal males but not in normal females or fibromyalgia patients Pain 2003 101 167 174 10.1016/S0304-3959(02)00325-1 12507711

[B41] Arendt-Nielsen L Sluka KA Nie HL Experimental muscle pain impairs descending inhibition Pain 2008 140 465 471 10.1016/j.pain.2008.09.027 18977598 PMC2732020

[B42] Tousignant-Laflamme Y Marchand S Excitatory and inhibitory pain mechanisms during the menstrual cycle in healthy women Pain 2009 146 47 55 10.1016/j.pain.2009.06.018 19592167

[B43] Baliki MN Geha PY Jabakhanji R Harden N Schnitzer TJ Apkarian AV A preliminary fMRI study of analgesic treatment in chronic back pain and knee osteoarthritis Mol Pain 2008 4 47 10.1186/1744-8069-4-47 18950528 PMC2584040

[B44] Kulkarni B Bentley DE Elliott R Julyan PJ Boger E Watson A Boyle Y El-Deredy W Jones AK Arthritic pain is processed in brain areas concerned with emotions and fear Arthritis Rheum 2007 56 1345 1354 10.1002/art.22460 17393440

[B45] Parks EL Geha PY Baliki MN Katz J Schnitzer TJ Apkarian AV Brain activity for chronic knee osteoarthritis: Dissociating evoked pain from spontaneous pain Eur J Pain 2011 15 843e1 843e14 21315627 10.1016/j.ejpain.2010.12.007 PMC3113642

[B46] Gwilym SE Filippini N Douaud G Carr AJ Tracey I Thalamic atrophy associated with painful osteoarthritis of the hip is reversible after arthroplasty: a longitudinal voxel-based morphometric study Arthritis Rheum 2010 62 2930 2940 10.1002/art.27585 20518076

[B47] Apkarian AV Bushnell MC Treede RD Zubieta JK Human brain mechanisms of pain perception and regulation in health and disease Eur J Pain 2005 9 463 484 10.1016/j.ejpain.2004.11.001 15979027

[B48] Gwilym SE Keltner JR Warnaby CE Carr AJ Chizh B Chessell I Tracey I Psychophysical and functional imaging evidence supporting the presence of central sensitization in a cohort of osteoarthritis patients Arthritis Rheum 2009 61 1226 1234 10.1002/art.24837 19714588

[B49] DeSantana JM Sluka KA Central mechanisms in the maintenance of chronic widespread noninflammatory muscle pain Curr Pain Headache Rep 2008 12 338 343 10.1007/s11916-008-0057-7 18765138 PMC2744440

[B50] Fillingim RB Gear RW Sex differences in opioid analgesia: clinical and experimental findings Eur J Pain 2004 8 413 425 10.1016/j.ejpain.2004.01.007 15324773

[B51] Chakrabarti S Liu NJ Gintzler AR Formation of mu-/kappa-opioid receptor heterodimer is sex-dependent and mediates female-specific opioid analgesia Proc Natl Acad Sci USA 2010 107 20115 20119 10.1073/pnas.1009923107 21041644 PMC2993367

[B52] Bodnar RJ Kest B Sex differences in opioid analgesia, hyperalgesia, tolerance and withdrawal: central mechanisms of action and roles of gonadal hormones Horm Behav 2010 58 72 81 10.1016/j.yhbeh.2009.09.012 19786031

[B53] Craft RM Sex differences in analgesic, reinforcing, discriminative, and motoric effects of opioids Exp Clin Psychopharmacol 2008 16 376 385 18837634 10.1037/a0012931

[B54] Dahan A Kest B Waxman AR Sarton E Sex-specific responses to opiates: animal and human studies Anesth Analg 2008 107 83 95 10.1213/ane.0b013e31816a66a4 18635471

[B55] Sorge RE LaCroix-Fralish ML Tuttle AH Sotocinal SG Austin JS Ritchie J Chanda ML Graham AC Topham L Beggs S Salter MW Mogil JS Spinal cord Toll-like receptor 4 mediates inflammatory and neuropathic hypersensitivity in male but not female mice J Neurosci 2011 31 15450 15454 10.1523/JNEUROSCI.3859-11.2011 22031891 PMC3218430

[B56] Ji Y Murphy AZ Traub RJ Estrogen modulates the visceromotor reflex and responses of spinal dorsal horn neurons to colorectal stimulation in the rat J Neurosci 2003 23 3908 3915 12736360 10.1523/JNEUROSCI.23-09-03908.2003 PMC6742189

[B57] Ji Y Tang B Cao DY Wang G Traub RJ Sex differences in spinal processing of transient and inflammatory colorectal stimuli in the rat Pain 2012 153 1965 1973 10.1016/j.pain.2012.06.019 22819535 PMC3413769

[B58] Lawson KP Nag S Thompson AD Mokha SS Sex-specificity and estrogen-dependence of kappa opioid receptor-mediated antinociception and antihyperalgesia Pain 2010 151 806 815 10.1016/j.pain.2010.09.018 20926192 PMC2972410

[B59] Mogil JS Bailey AL Sex and gender differences in pain and analgesia Prog Brain Res 2010 186 141 157 21094890 10.1016/B978-0-444-53630-3.00009-9

[B60] Loyd DR Wang X Murphy AZ Sex differences in μ-opioid receptor expression in the rat midbrain periaqueductal gray are essential for eliciting sex differences in morphine analgesia J Neurosci 2008 28 14007 14017 10.1523/JNEUROSCI.4123-08.2008 19109484 PMC2819468

[B61] Ge HY Madeleine P Arendt-Nielsen L Gender differences in pain modulation evoked by repeated injections of glutamate into the human trapezius muscle Pain 2005 113 134 140 10.1016/j.pain.2004.09.041 15621373

[B62] Loyd DR Murphy AZ Sex differences in the anatomical and functional organization of the periaqueductal gray-rostral ventromedial medullary pathway in the rat: a potential circuit mediating the sexually dimorphic actions of morphine J Comp Neurol 2006 496 723 738 10.1002/cne.20962 16615128 PMC2823481

[B63] Lopez-Olivo MA Landon GC Siff SJ Edelstein D Pak C Kallen MA Stanley M Zhang H Robinson KC Suarez-Almazor ME Psychosocial determinants of outcomes in knee replacement Ann Rheum Dis 2011 70 1775 1781 10.1136/ard.2010.146423 21791452

[B64] Borkhoff CM Hawker GA Wright JG Patient gender affects the referral and recommendation for total joint arthroplasty Clin Orthop Relat Res 2011 469 1829 1837 10.1007/s11999-011-1879-x 21448775 PMC3111793

[B65] Vranceanu AM Barsky A Ring D Psychosocial aspects of disabling musculoskeletal pain J Bone Joint Surg Am 2009 91 2014 2018 10.2106/JBJS.H.01512 19651964

[B66] Perrot S Poiraudeau S Kabir M Bertin P Sichere P Serrie A Rannou F Active or passive pain coping strategies in hip and knee osteoarthritis? Results of a national survey of 4,719 patients in a primary care setting Arthritis Rheum 2008 59 1555 1562 10.1002/art.24205 18975370

[B67] Unruh AM Gender variations in clinical pain experience Pain 1996 65 123 167 10.1016/0304-3959(95)00214-6 8826503

[B68] Riddle DL Wade JB Jiranek WA Kong X Preoperative pain catastrophizing predicts pain outcome after knee arthroplasty Clin Orthop Relat Res 2010 468 798 806 10.1007/s11999-009-0963-y 19585177 PMC2816776

[B69] Pells JJ Shelby RA Keefe FJ Dixon KE Blumenthal JA Lacaille L Tucker JM Schmitt D Caldwell DS Kraus VB Arthritis self-efficacy and self-efficacy for resisting eating: relationships to pain, disability, and eating behavior in overweight and obese individuals with osteoarthritic knee pain Pain 2008 136 340 347 10.1016/j.pain.2007.07.012 17764844 PMC2494734

[B70] Shelby RA Somers TJ Keefe FJ Pells JJ Dixon KE Blumenthal JA Domain specific self-efficacy mediates the impact of pain catastrophizing on pain and disability in overweight and obese osteoarthritis patients J Pain 2008 9 912 919 10.1016/j.jpain.2008.05.008 18602871 PMC2581839

[B71] Fransen M McConnell S Bell M Therapeutic exercise for people with osteoarthritis of the hip or knee. A systematic review. J Rheumatol 2002 29 1737 1745 12180738

[B72] Somers TJ Keefe FJ Pells JJ Dixon KE Waters SJ Riordan PA Blumenthal JA McKee DC Lacaille L Tucker JM Schmitt D Caldwell DS Kraus VB Sims EL Shelby RA Rice JR Pain catastrophizing and pain-related fear in osteoarthritis patients: relationships to pain and disability J Pain Symptom Manage 2009 37 863 872 10.1016/j.jpainsymman.2008.05.009 19041218 PMC2702756

[B73] Tsai YF Gender differences in pain and depressive tendency among Chinese elders with knee osteoarthritis Pain 2007 130 188 194 10.1016/j.pain.2007.03.014 17452080

[B74] Racine M Tousignant-Laflamme Y Kloda LA Dion D Dupuis G Choiniere M A systematic literature review of 10 years of research on sex/gender and pain perception - part 2: do biopsychosocial factors alter pain sensitivity differently in women and men? Pain 2012 153 619 635 10.1016/j.pain.2011.11.026 22236999

